# Emerging trends and hotspots in peptic ulcer from 2008 to 2023: A bibliometric analysis

**DOI:** 10.1097/MD.0000000000039557

**Published:** 2024-09-06

**Authors:** Jiahui Li, Jiamei Jin, Xiaoyang Wang, Fugang Huang, Ke Sun, Menglin Li, Xiaogu Liu

**Affiliations:** a Haiyan Hospital of Traditional Chinese Medicine, Jiaxing, China; b The Basic Medical College of Zhejiang Chinese Medical University, Hangzhou, China; c First School of Clinical Medicine, Zhejiang Chinese Medical University, Hangzhou, China.

**Keywords:** bibliometric, citespace, peptic ulcer, VOSviewer

## Abstract

Peptic ulcer (PU) is a common digestive disorder in the gastroduodenal. Although bibliometrics has become very popular in the medical field, a bibliometric analysis of research related to PU has yet to be reported. Therefore, this research aims to analyze the trends and hotspots of PU in the last 15 years. Literature data related to PU retrieved from the Web of Science Core Collection database from 2008 to 2023 were visualized and analyzed using CiteSpace 6.1.6.msi, VOSviewer 1.6.19, and SCImago Graphica Beta 1.0.35. Six thousand four hundred ninety-one papers were collected based on inclusion and exclusion criteria. The country with the highest number of publications was China. The institution with the highest number of publications was Baylor College of Medicine. The most prolific author was Yamaoka Yoshio. Malfertheiner Peter had the highest number of citations. The journal with the most publications is World Journal of Gastroenterology. The most cited Journal is Gastroenterology. The most cited reference was published by Marshall B. J. et al in 1984. The article with the highest burst strength was published in 2012 by Malfertheiner Peter. The keyword with the highest burst strength was “oxidative stress.” Our research provides a bibliometric analysis of PU research to reveal the trends and hotspots of PU for 2008 to 2023. Our findings will help researchers to quickly understand the current state of research and provide a reference for in-depth studies in this area to foster the development of PU research.

## 1. Introduction

Peptic ulcer (PU) is a break of >3 to 5 mm in the mucosa of the stomach or duodenum with a visible depth.^[1]^ The clinical manifestations of PU are epigastric pain, nausea, early satiety, bloating, belching, or postprandial discomfort.^[1,2]^ Complications include bleeding, perforation, penetration, and gastric outlet obstruction,^[3]^ with *Helicobacter pylori* (*H pylori*) and nonsteroidal anti-inflammatory drugs (NSAIDs) as significant risk factors.^[4,5]^ PU affects 4 million people worldwide each year, with an estimated lifetime prevalence of 5% to 10% in the general population.^[6]^

Bibliometrics can analyze the literature in a particular field to understand its knowledge structure and explore its development trend. It can be combined with CiteSpace, VOSviewer, and other visual analysis software to show the current research status and hotspots of a specific field in the form of images, which explore the relationship between countries, institutions, authors, journals, cited literature, and keywords.^[7]^ It has been applied to the research of cancer,^[8]^ cardiovascular system diseases,^[9]^ and endocrine system diseases.^[10]^ However, there is no bibliometric analysis study on PU. Therefore, this study systematically analyzes the studies on PU between 2008 and 2023 and evaluate the current status and trends of related research.

## 2. Materials and methods

We searched the Web of Science Core Collection for Science Citation Index Expanded (SCI-Expanded) and downloaded the data on June 23, 2023. Search terms included: TS = (Gastroduodenal Ulcer OR Peptic Ulcer). Limit publication time to from January 1, 2008 to May 13, 2023, and the document type was restricted to thesis and review papers, excluding non-English literature. We ended up with 6491 articles and reviews that met the inclusion criteria for analysis.

These “fully documented and cited references” documents were extracted into CiteSpace 6.1.6.msi and VOSviewer 1.6.19 in “plain text file” format to identify the principal countries, institutions, authors, journals, references, and keywords. The resulting information was visualized and analyzed using CiteSpace 6.1.6.msi, VOSviewer 1.6.19, and SCImago Graphica Beta 1.0.35.

## 3. Results

### 3.1. Annual numbers of publications

**Figure 1.**
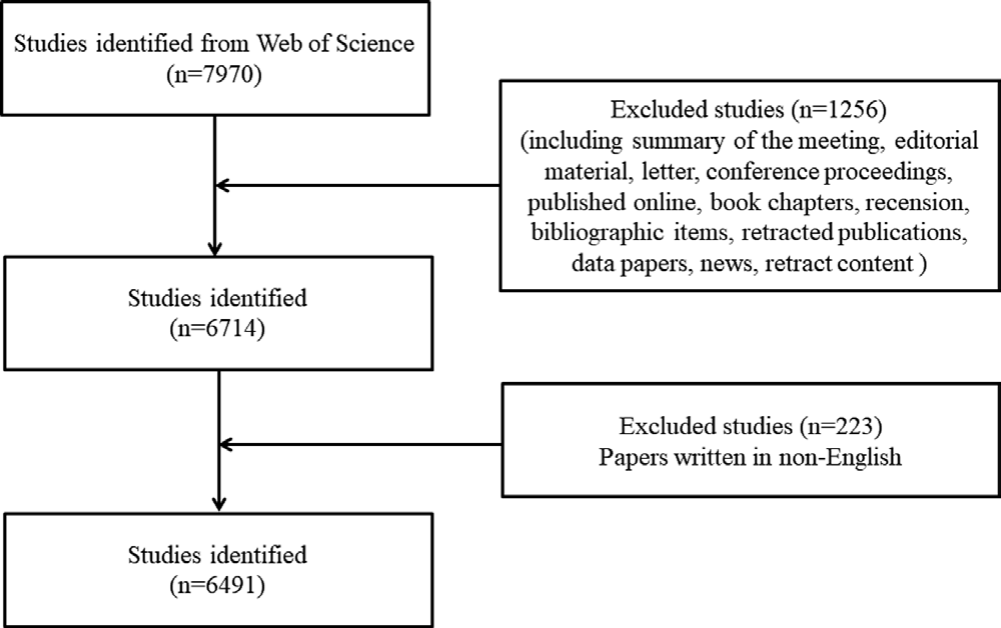
Detailed process for literature screening.

As shown in Figure 1, 7970 papers related to PU were published between January 01, 2008 and May 13, 2023. According to the inclusion and exclusion criteria, a total of 6491 papers were obtained, which were written by 30,240 authors from 6644 institutions in 132 countries, published in 1554 journals, and cited 155,158 times by 19,443 journals.

**Figure 2.**
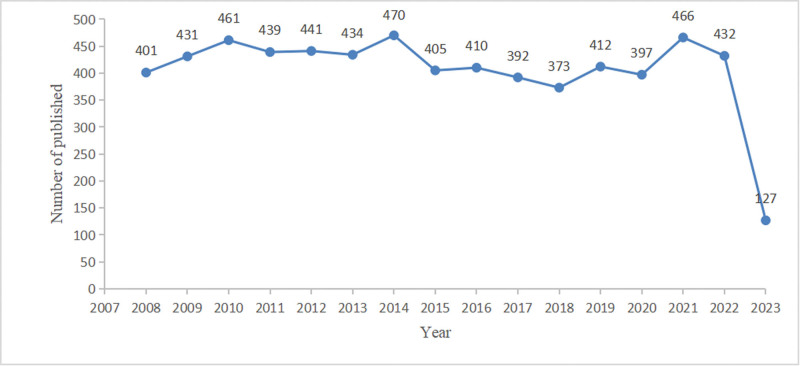
Trend of publications in the field of peptic ulcer (2008–2023).

As shown in Figure 2, the number of papers published from 2008 to 2022 remained steady at more than 370. The lowest in 2018 (n = 373) and peaked in 2014 (n = 470), indicating that PU has always been on the minds of scholars.

### 3.2. Analysis of countries

**Figure 3.**
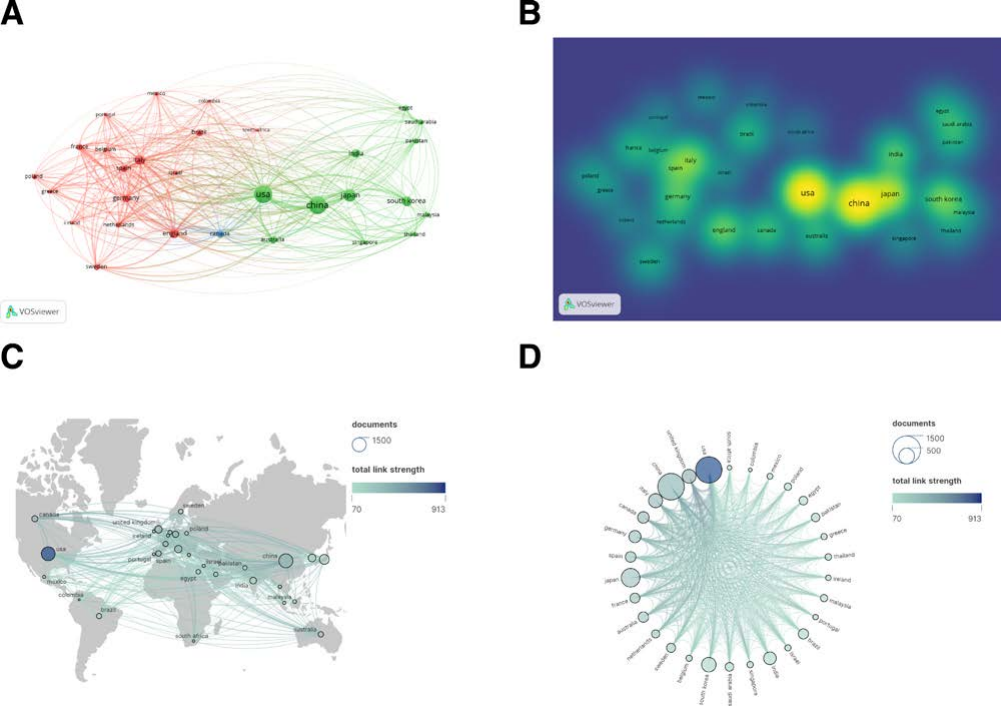
Top 30 countries with research contributions to peptic ulcer. (A) Country collaboration map. (B) Countries’ density map. (C) World map of countries’ cooperation density. (D) A circle diagram evaluating the international collaboration between clusters.

The study utilizes VOSviewer and Scimago Graphica for country visualization and analysis. Among the top 10 countries ranked in terms of the number of publications, China has the highest number of publications (1252 publications) but a low average number of citations, followed by the United States (1235 publications) and Japan (620 publications). In addition, the United Kingdom, Germany, and Italy had the highest average citations, indicating a high level of scientific research in European countries (Fig. 3A and B; Table 1). The country with the highest number of collaborations with other countries is the United States, followed by the United Kingdom and China. The lines in the graph indicate collaboration, and the darker the color, the more collaboration with other countries, the way most countries have light colors, indicating that these countries do not have stable communication and collaboration (Fig. 3C and D).

**Table 1:** Top 10 countries related to PU.

Rank	Countries	Count	Citations	Average citations
1	China	1252	24,062	19.22
2	USA	1235	51,209	41.46
3	Japan	620	17,276	27.86
4	South Korea	375	6996	18.66
5	Italy	350	15,551	44.43
6	England	342	17,755	51.92
7	Iran	296	6070	20.51
8	India	288	7093	24.63
9	Germany	261	13,401	51.34
10	Canada	235	11,003	46.82

PU = peptic ulcer.

### 3.3. Analysis of institutions

**Figure 4.**
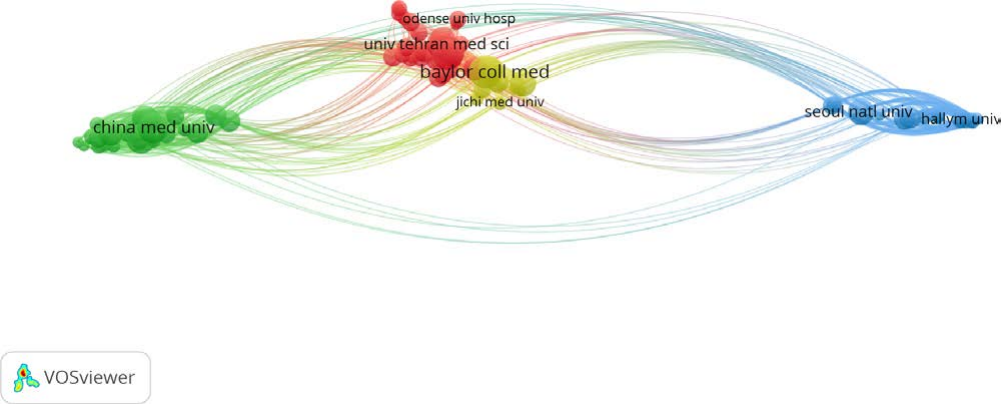
Top 100 institutions with research contributions to peptic ulcer.

VOSviewer was used to map the visualization of the institutions. The top 10 institutions in terms of publications are shown in Table 2, with Baylor Coll Med (108 publications) having the highest number of publications, followed by Chinese Univ Hong Kong (97 publications) and China Med Univ (95 publications). 7/10 of the institutions in Asia. However, the United States institutions (Baylor Coll Med and Michael E DeBakey VA Med Ctr) have the highest average citations, with the top 100 most published institutions centered on Baylor Coll Med (Fig. 4).

**Table 2:** Top 10 institutions related to PU.

Rank	Institutions	Countries	Count	Citations	Average citations
1	Baylor Coll Med	USA	108	6539	60.55
2	Chinese Univ Hong Kong	China	97	5478	56.47
3	China Med Univ	China	95	1756	18.48
4	Natl Yang Ming Univ	China	92	2097	22.79
5	Seoul Natl Univ	South Korea	72	1898	26.36
6	Oita Univ	Japan	69	2483	35.99
7	Chang Gung Univ	China	68	738	10.85
8	Univ Tehran Med Sci	Iran	68	1462	21.50
9	Michael E DeBakey VA Med Ctr	USA	65	4500	69.23
10	Mcgill Univ	Canada	63	1827	29.00

PU = peptic ulcer.

### 3.4. Analysis of authors and cited authors

**Figure 5.**
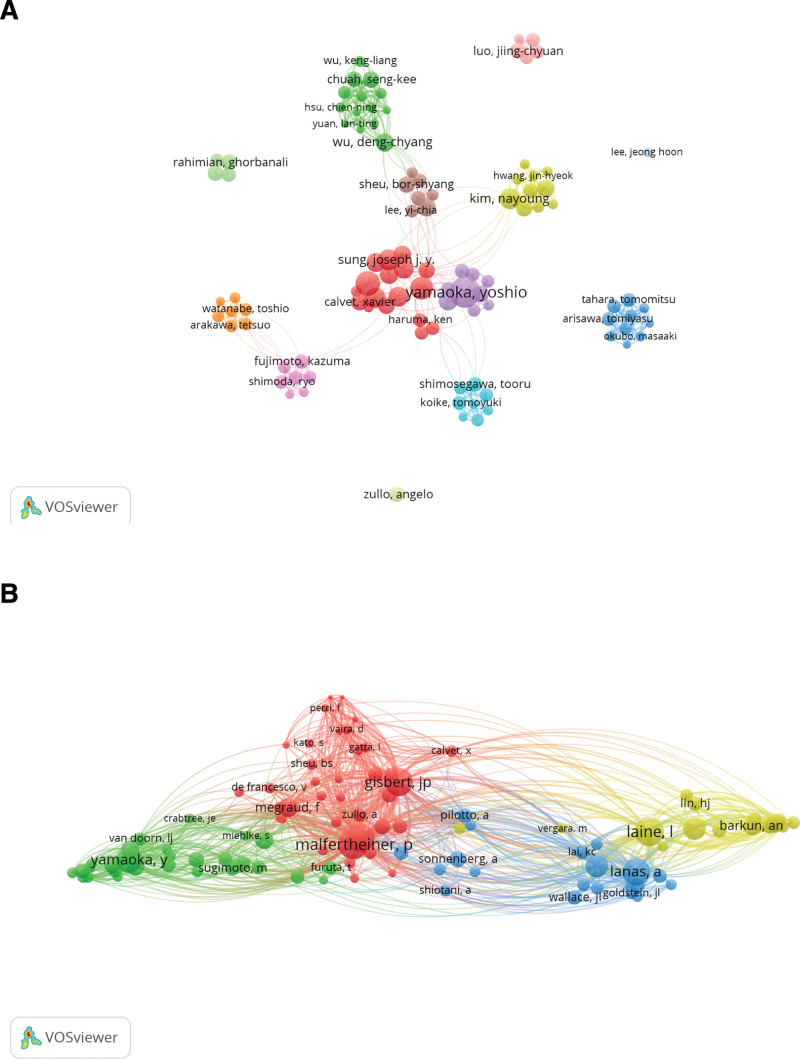
(A) Top 100 authors with research contributions to peptic ulcer. (B) Top 100 cited authors with research contributions to peptic ulcer.

The top 10 authors in terms of publications ranked Yamaoka Yoshio (68 publications) first, followed by Lanas Angel (42 publications) and Graham David Y. (35 publications). Malfertheiner Peter (126.90 average citations) had the highest average number of citations, followed by Graham David Y. (119.06 average citations) and Chan Francis K. L. (117.21 average citations) (Fig. 5A; Table 3). Among the top 10 co-cited authors ranked in terms of paper citations, Malfertheiner Peter has the most citations, with 5 out of 30,240 co-cited authors having been cited more than 1000 times, which suggests that their scholarship is recognized and cited by the majority of scholars (Fig. 5B; Table 4).

**Table 3:** Top 10 authors related to PU.

Rank	Authors	Count	Citations	Average citations
1	Yamaoka, Yoshio^[11]^	68	2860	42.06
2	Lanas, Angel^[12]^	42	3602	85.76
3	Graham, David Y.	35	4167	119.06
4	Kim, Nayoung	31	1092	35.23
5	Sung, Joseph J. Y.	30	1726	57.53
6	Chan, Francis K. L.	29	3399	117.21
7	Gisbert, Javier P.	29	2527	87.14
8	Malfertheiner, Peter	29	3680	126.90
9	Barkun, Alan N.	27	908	33.63
10	Sugimoto, Mitsushige^[13]^	26	807	31.04

PU = peptic ulcer.

**Table 4:** Top 10 cited authors related to PU.

Rank	Cited authors	Citations
1	Malfertheiner, Peter	1366
2	Laine, Loren	1250
3	Graham, David Y.	1234
4	Gisbert, Javier P.	1217
5	Lanas, Angel^[12]^	1157
6	Yamaoka, Yoshio^[11]^	961
7	Chan, Francis K. L	763
8	Sung, Joseph J. Y.	761
9	Megraud, Francis	542
10	Barkun, Alan N.	535

PU = peptic ulcer.

### 3.5. Analysis of journals and cited journals

**Figure 6.**
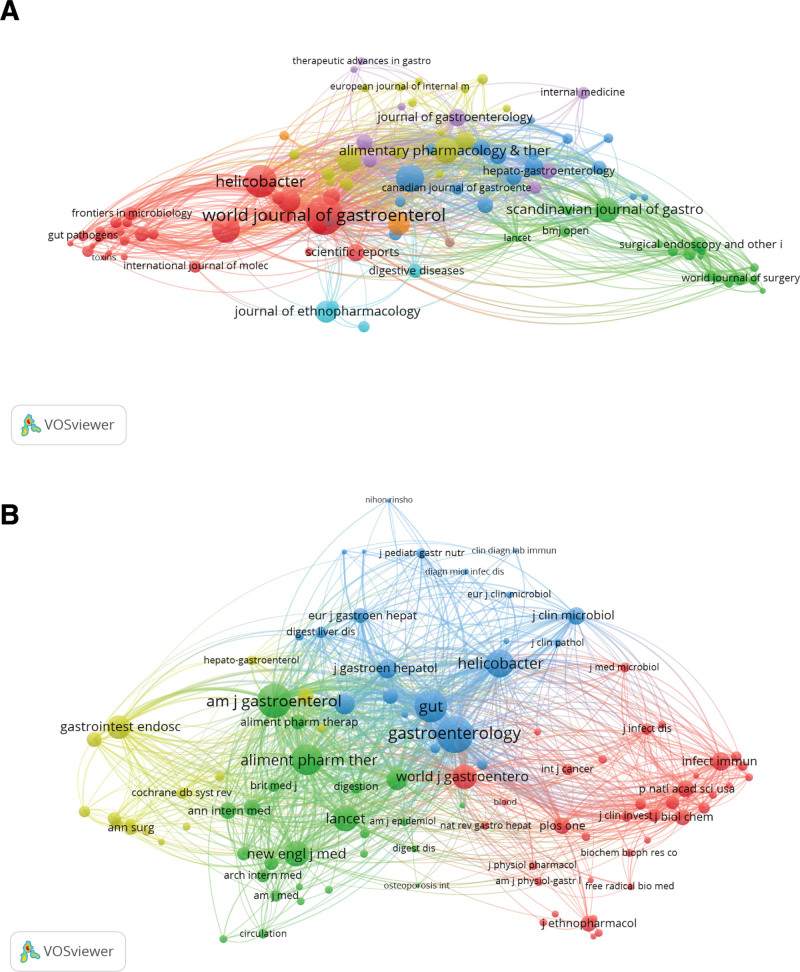
(A) Top 100 journals with research contributions to peptic ulcer. (B) Top 100 cited journals with research contributions to peptic ulcer.

Table 5 lists the top 10 journals ranked in terms of the number of papers published related to PU, with an average impact factor of 4.46. These journals accept the most significant number of articles related to PU and are recommended to be followed by scholars researching PU. World Journal of Gastroenterology (223 publications) is the most prolific journal in this field, followed by Helicobacter (157 publications) and Plos One (121 publications) (Fig. 6A; Table 5). Impact factor (IF) is an internationally recognized journal evaluation metric. Among the top 10 journals selected, Alimentary Pharmacology & Therapeutics (IF = 9.524) has the highest impact factor and average citation frequency. Table 6 shows the top 10 cited journals related to PU. Most of these magazines are Q1 in the Journal Citation Reports. The most cited journal was Gastroenterology (10,831 citations), followed by American Journal of Gastroenterology (9219 citations) and Gut (8402 citations) (Fig. 6B; Table 6). The higher average impact factor (IF = 49.05) was due to the high impact factors of Lancet (IF = 202.731) and New England Journal of Medicine (IF = 176.082). World Journal of Gastroenterology, Helicobacter, Alimentary Pharmacology & Therapeutics, and Digestive Diseases and Sciences are present in Tables 5 and 6, indicating that these journals have received wide attention in gastroenterology.

**Table 5:** Top 10 journals related to PU.

Rank	Journals	Count	IF (2021)	JCR	Citations	Average citations
1	World Journal of Gastroenterology	223	5.374	Q2	6464	28.99
2	Helicobacter	157	5.182	Q2	4130	26.31
3	Plos One	121	3.752	Q2	3096	25.59
4	Digestive Diseases and Sciences	118	3.487	Q3	1907	16.16
5	Alimentary Pharmacology & Therapeutics	97	9.524	Q1	3988	41.11
6	Scandinavian Journal of Gastroenterology	91	3.027	Q4	1305	14.34
7	Journal of Gastroenterology and Hepatology	84	4.369	Q2	2308	27.48
8	BMC Gastroenterology	78	2.848	Q4	1260	16.15
9	Medicine	76	1.817	Q3	419	5.51
10	Journal of Ethnopharmacology	72	5.195	Q2	1884	26.17

IF = impact factor, JCR = journal citation reports, PU = peptic ulcer.

**Table 6:** Top 10 cited journals related to PU.

Rank	Cited journals	Citations	IF (2021)	JCR
1	Gastroenterology	10,831	33.883	Q1
2	American Journal of Gastroenterology	9219	12.045	Q1
3	Gut	8402	31.795	Q1
4	Alimentary Pharmacology & Therapeutics	7326	9.524	Q1
5	Helicobacter	6216	5.182	Q2
6	Lancet	5227	202.731	Q1
7	New England Journal of Medicine	5051	176.082	Q1
8	World Journal of Gastroenterology	4781	5.374	Q2
9	Gastrointestinal Endoscopy	4356	10.396	Q1
10	Digestive Diseases and Sciences	3688	3.487	Q3

IF = impact factor, JCR = journal citation reports, PU = peptic ulcer.

### 3.6. Analysis of references

**Figure 7.**
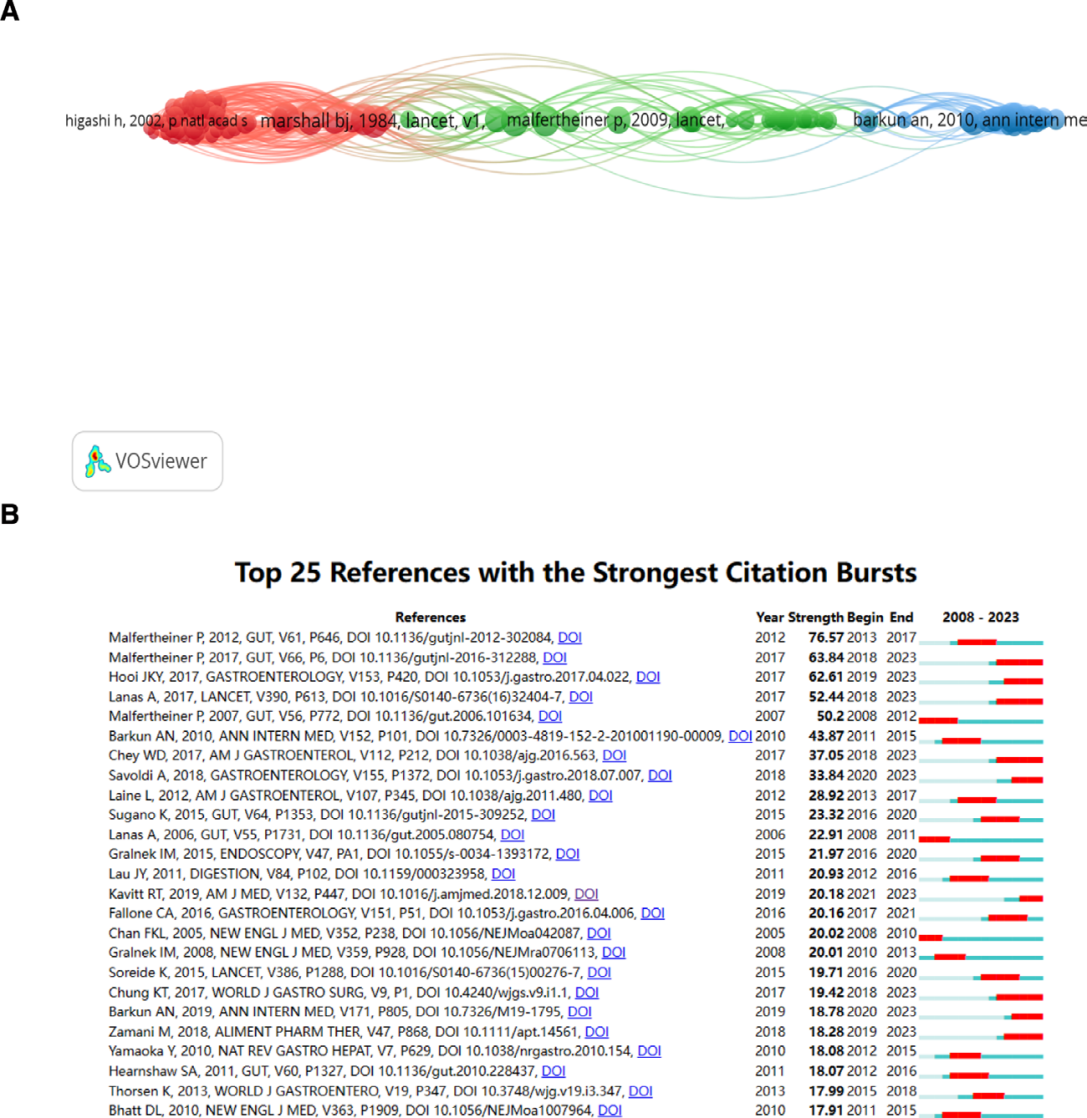
(A) Top 100 cited references with research contributions to peptic ulcer. (B) Top 25 cited references with the strongest citation bursts.

Citespace and VOSviewer were used to map the visualization of the references. The top 10 references cited in PU research were all published between 1984 and 2012. The most cited reference is “Unidentified curved bacilli in the stomach of patients with gastritis and peptic ulceration.” by Marshall, B. J. et al 1984, which was cited 301 times (Fig. 7A; Table 7). Based on the burst detection analysis of the references, the results of the first 25 articles are shown in Figure 7B. Burst detection identifies articles that are intriguing to people in a specific period. The article with the highest burst strength was published in 2012 by Malfertheiner Peter in Gut: Management of *Helicobacter pylori* infection-the Maastricht IV/ Florence Consensus Report.

**Table 7:** Top 10 cited references related to PU.

Rank	Cited references	Authors	Year	Citations
1	Unidentified curved bacilli in the stomach of patients with gastritis and peptic ulceration.	Marshall, B J^[14]^	1984	301
2	Classification and grading of gastritis. The updated Sydney System. International Workshop on the Histopathology of Gastritis, Houston 1994	Dixon, M F	1996	280
3	Management of *Helicobacter pylori* infection-the Maastricht IV/ Florence Consensus Report	Malfertheiner, P^[15]^	2012	262
4	Current concepts in the management of *Helicobacter pylori* infection: the Maastricht III Consensus Report.	Malfertheiner, P^[16]^	2007	259
5	International Consensus Recommendations on the Management of Patients With Nonvariceal Upper Gastrointestinal Bleeding	Barkun, AN	2010	250
6	Risk assessment after acute upper gastrointestinal hemorrhage	Rockall, TA	1996	238
7	*Helicobacter pylori* infection.	Suerbaum, S	2002	234
8	*Helicobacter pylori* infection and the development of gastric cancer.	Uemura, N	2001	231
9	Pathogenesis of *Helicobacter pylori* infection	Kusters, J G	2006	219
10	Mosaicism in vacuolating cytotoxin alleles of *Helicobacter pylori*. Association of specific vacA types with cytotoxin production and peptic ulceration.	Atherton, J C	1995	201

PU = peptic ulcer.

### 3.7. Analysis of keywords

**Figure 8.**
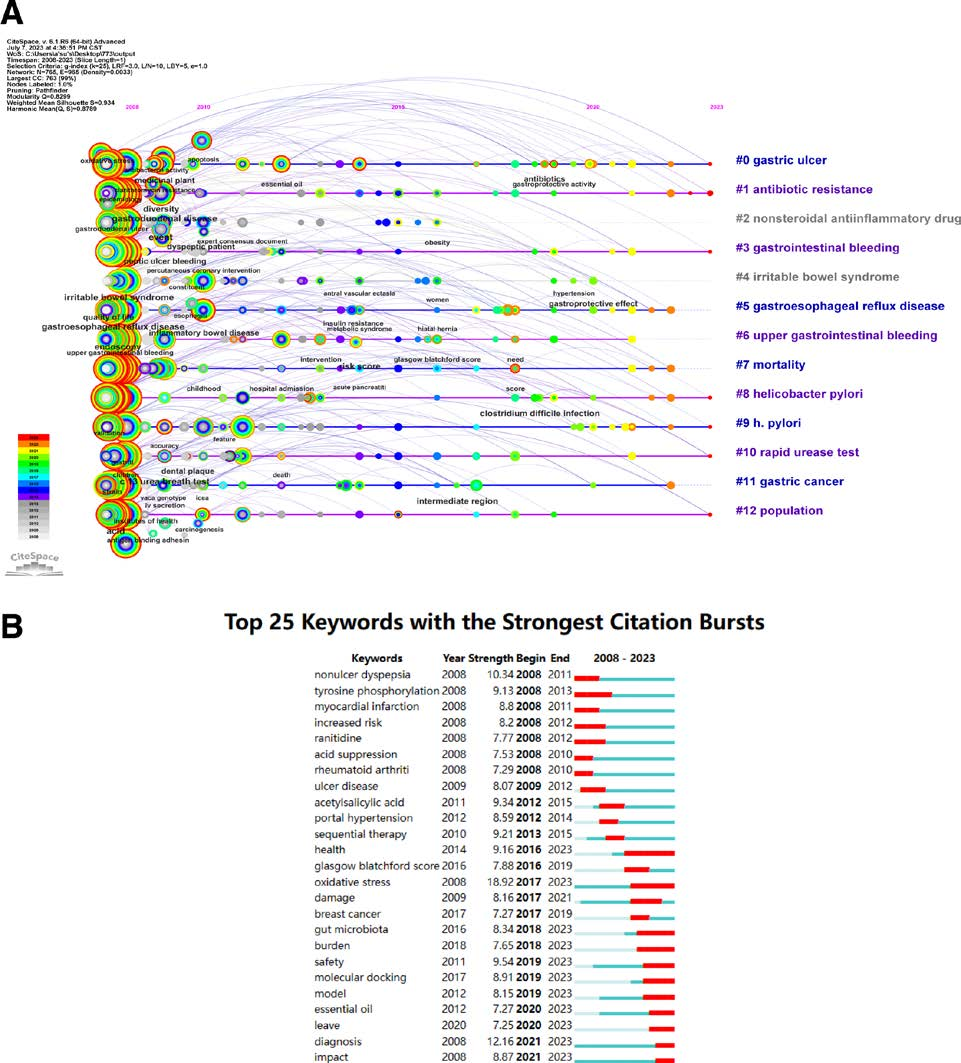
(A) Timeline view of keywords related to peptic ulcer. (B) Top 25 keywords with the strongest citation bursts.

Citespace was used to map the visualization of keywords. Keywords can reflect the hotspots and frontiers of a specific field. Seven hundred sixty-five keywords were identified for PU research, and the top 10 most frequent keywords are shown in Table 8. The most common keywords are peptic ulcer (1867 times), *H pylori* (1775 times), and infection (840 times), the highest centrality of the keywords were gastroesophageal reflux disease (0.05), *H pylori* eradication (0.05), injury (0.05), inflammatory bowel disease (0.05), and stress (0.05). Figure 8A shows a timeline plot of the keywords, which categorizes into 13 large clusters: “gastric ulcer,” “antibiotic resistance,” “nonsteroidal antiinflammatory drug,” “gastrointestinal bleeding,” “irritable bowel syndrome,” “gastroesophageal reflux disease,” “upper gastrointestinal bleeding,” “mortality,” “helicobacter pylori,” “h.pylori,” “rapid urease test,” “gastric cancer,” and “population.” Figure 8B shows the burst detection analysis of keywords from 2008 to 2023, where the blue line is the timeline and the red line is the period of the outbreaks, reflecting the changes in research hotspots over time. From 2008 to 2012, the research hotspots of PU were “nonulcer dyspepsia,” “tyrosine phosphorylation,” “myocardial infarction,” “increased risk,” “ranitidine,” “acid suppression,” “rheumatoid arthriti,” and “ulcer disease.” “Acetylsalicylic acid,” “portal hypertension,” and “sequential therapy” broke out from 2012 to 2015. From 2016 to 2023, “health,” “glasgow blatchford score,” “oxidative stress,” “damage,” “breast cancer,” “gut microbiota,” “burden,” “safety,” “molecular docking,” “model,” “essential oil,” “leave,” “diagnosis,” and “impact” are the research hotspots for PU. The keyword with the highest strength is “oxidative stress,” with a score of 18.92, and the second is “diagnosis” with a score of 12.16, both hotspots from 2016 to 2023. In addition, “health,” “oxidative stress,” “gut microbiota,” “burden,” “safety,” “molecular docking,” “model,” “essential oil,” “leave,” “diagnosis,” and “impact” are still of concern to scholars.

**Table 8:** Top 10 keywords related to PU.

Rank	Keywords	Count	Keywords	Centrality
1	Peptic ulcer	1867	Gastroesophageal reflux disease	0.05
2	*Helicobacter pylori*	1775	*Helicobacter pylori* eradication	0.05
3	Infection	840	Injury	0.05
4	Peptic ulcer disease	747	Inflammatory bowel disease	0.05
5	Management	703	Stress	0.05
6	Risk	632	Randomized controlled trial	0.04
7	Proton pump inhibitor	609	Intestinal metaplasia	0.04
8	Disease	608	Gastric mucosa	0.04
9	Prevalence	571	Colonization	0.04
10	*Helicobacter pylori* infection	558	Esomeprazole	0.04

PU = peptic ulcer.

## 4. Discussion

### 4.1. General information

We screened a total of 6491 papers on PU from Web of Science Core Collection, and the number of papers has steadily increased during this period of 15 years. China has the most significant number of papers published and has made outstanding contributions to PU research. In addition, 4 of the top 10 research institutions are from China, including Chinese Univ Hong Kong, China Med Univ, Natl Yang Ming Univ, and Chang Gung Univ, which indicates that Chinese institutions have paid much attention to PU research in recent years. The United States has the second most papers published, only 17 papers less than the number of papers published in China. However, the average citation in the United States is very high. Although only Baylor Coll Med and Michael E DeBakey VA Med Ctr institutions in the top ten rankings belong to the United States, they are the 2 institutions with the highest average citation, which indicates that the quality of the papers in the United States is very high. In addition, the United States has carried out extensive cooperation in this field, which is much higher than other countries, so other countries should pay more attention to interregional cooperation and exchange.

Yamaoka Yoshio is the author with the most publications in this field and is primarily involved in the pathogenesis of gastroduodenal diseases associated with *H pylori*.^[11,13,17,18]^ Malfertheiner Peter is the author of the most cited papers in this field and is primarily involved in developing the management of *H pylori* infections, one of the causes of PU.^[15,16,19]^ World Journal of Gastroenterology is the most included journal, with an IF of 5.374. The journal is published in the United States and focuses on articles in gastroenterology and hepatology. Gastroenterology is the most cited journal, with an IF of 33.883. The journal is published in the United States and focuses on articles in the field of gastroenterology. It is a leading journal in the field of gastrointestinal disease. Marshall B. J. et al published the most cited article, “Unidentified curved bacilli in the stomach of patients with gastritis and peptic ulceration.” This study found that gastritis and PU may be caused by *H pylori* infection,^[14]^ which was confirmed in subsequent studies for which they were awarded the Nobel Prize in Physiology or Medicine in 2005. It is worth noting that 7 of the 10 most cited papers were on *H pylori*, which suggests that *H pylori* has a significant role in PU. The article with the highest intensity of blasts was published by Malfertheiner P et al “Management of *Helicobacter pylori* infection-the Maastricht IV/Florence Consensus Report.” It discusses the management of *H pylori* infection and is the most recent of the 10 papers with the most cited papers, suggesting that *H pylori* continues to be a hot research topic in recent years.

### 4.2. Hotspots and frontiers

Combining the frequency of keywords, timeline mapping, and burst word mapping, we summarized the research hotspots and development directions in the field of PU.

#### 4.2.1. Pathogenic mechanisms and risk factors of PU

*H pylori* and the use of NSAIDs are major risk factors for PU.^[20]^

*H pylori* is a gram-negative, spiral-shaped, microaerobic bacterium that colonizes the human gastric mucosa. *H pylori* infection is closely associated with PU, and bacterial and host factors mediate its pathogenesis. *H pylori* utilizes urease to neutralize the stomach’s acidic environment^[21,22]^ and flagella to regulate motility towards the gastric epithelium^[23]^ and successfully colonize and establish a persistent infection. It secretes outer membrane proteins, including blood group antigen-binding adherence and sialic acid-binding adherence, which interact with antigens in the gastroduodenal tract to promote bacterial adhesion to the gastric epithelium,^[24]^ and finally release a variety of virulence factors, including cytotoxin-associated gene A and vacuolating cytotoxin A, leading to gastric tissue damage.^[25,26]^ *H pylori* induces chronic inflammation in the gastric mucosa mediated by a range of pro- and anti-inflammatory cytokines, and host gene polymorphisms can cause PU by influencing inter-individual differences in the degree of cytokine response, leading to an imbalance between attack and defense factors in the mucosa. Several pro-inflammatory cytokines (IL-1B, IL-6, IL-8, and TNF-α) and anti-inflammatory cytokines (IL-10) are associated with PU.^[27]^

NSAIDs are commonly used to treat inflammatory pain. The mechanisms by which NSAIDs produce damage to the gastroduodenal can be categorized into local and systemic actions, including the induction of injury by 3 key pathways: inhibition of cyclooxygenase-1 activity, inhibition of cyclooxygenase-2 activity, and direct cytotoxicity effects on the epithelium.^[28]^ Systemic inhibition of cyclooxygenase-1-derived prostaglandins is thought to be the primary mechanism. Reduced mucosal prostaglandin levels are associated with decreased mucus and bicarbonate secretion, inhibition of cell proliferation, and decreased mucosal blood flow, which is essential for maintaining mucosal integrity, therefore reduced mucosal prostaglandin levels lead to PU.^[12]^

#### 4.2.2. Diagnosis of PU

PU clinically manifests itself in epigastric pain, dyspepsia, and other symptoms, and endoscopy is the gold standard for diagnosing PU. Since *H pylori* is a significant cause of PU, examination of *H pylori* is essential. Diagnostic methods for *H pylori* infection can be categorized as invasive and noninvasive. Invasive testing involves a biopsy sample obtained during esophagogastroduodenoscopy, which is then subjected to histologic analysis, rapid urease assay, molecular methods, or culture to diagnose the presence of *H pylori* infection. Noninvasive tests include serologic testing, fecal antigen testing, 13C-urea breath testing, and so on.^[29]^

The American Society for Gastrointestinal Endoscopy recommends esophagogastroduodenoscopy for patients over 50 years of age suspected of having PU, and noninvasive testing for *H pylori* such as the 13C-urea breath testing and fecal antigen testing for patients under 50 years of age with no symptoms other than dyspepsia.^[30,31]^

#### 4.2.3. Treatment of PU

The eradication of *H pylori* is crucial as it is one of the main factors causing PU. Therapeutic regimens for *H pylori* eradication are based on a combination of strong acid inhibitors and antibiotics. The main clinical programs are proton pump inhibitor triple (PPI) therapy, bismuth quadruple therapy, and the resulting sequential, concomitant, and hybrid therapies.

PPI triple therapy (PPIs plus 2 antibiotics: clarithromycin, amoxicillin, or metronidazole) for 7 to 10 days used to be the standard of care and was the first-line treatment option for *H pylori* eradication at that time.^[32]^ PPIs inhibiting active parietal cell acid secretion are the drugs of choice for PU, *H pylori* infections, and for the prevention of NSAID-associated ulcers.^[33]^ With consistent patient tolerability, promising safety profile, and superior antacid capacity, PPIs are gradually becoming the mainstay of therapy for acid-related diseases.^[34]^ Clinical studies have shown that the 4-week cure rate of duodenal ulcers treated with the novel PPI anaprazole is 90.9%.^[35]^ However, the increasing prevalence of antibiotic-resistant strains has become increasingly ineffective. Therefore, tetracycline, which is more effective in eradication, is considered a first-line treatment.^[36]^

Bismuth quadruple therapy, which consists of PPIs, bismuth, tetracycline, and nitroimidazole, has been shown to significantly increase the eradication rates of clarithromycin, metronidazole, and dual-resistant strains by 40%, 26%, and 59%.^[37]^ The American College of Gastroenterology, Toronto Consensus recommends bismuth tetracycline for 10 to 14 days as a first-line treatment option because of its high eradication rate, low susceptibility to resistance, and high safety profile.^[32,38]^

Concomitant therapy was PPIs, amoxicillin, clarithromycin, and nitroimidazole therapy for 10 to 14 days. Sequential therapy was PPIs and amoxicillin treatment for the first 5 or 7 days and PPIs, clarithromycin, and nitroimidazole treatment for the next 5 or 7 days. Hybrid therapy is a mixture of sequential and concomitant therapy, with sequential therapy for the first 5 or 7 days and concomitant therapy for the next 5 or 7 days, i.e., PPIs and amoxicillin for the first 5 or 7 days and PPIs, amoxicillin, clarithromycin, and nitroimidazole for the next 5 or 7 days. All 3 have good efficacy against *H pylori*.^[32,38,39]^

## 5. Conclusion

This study is the first to systematically analyze the literature related to PU using bibliometric methods to provide insights for further research. In the past 15 years, global research on PU has remained hot. We should pay more attention to the papers published in professional journals. We should strengthen the cooperation among countries, institutions, and authors to study PU more deeply, and the research on the diagnosis and pathogenesis of PU is still the future research direction.

## Author contributions

**Funding acquisition:** Xiaogu Liu.

**Investigation:** Jiahui Li, Jiamei Jin, Ke Sun.

**Methodology:** Jiahui Li, Xiaoyang Wang.

**Software:** Jiahui Li, Fugang Huang.

**Supervision:** Xiaogu Liu.

**Validation:** Fugang Huang.

**Visualization:** Jiahui Li, Fugang Huang.

**Writing – original draft:** Jiahui Li.

**Writing – review & editing:** Jiamei Jin, Xiaoyang Wang, Menglin Li.
